# Mesorectal reconstruction with pedicled greater omental transplantation to relieve low anterior resection syndrome following total intersphincteric resection in patients with ultra-low rectal cancer

**DOI:** 10.1186/s12893-023-02140-1

**Published:** 2023-08-12

**Authors:** Jiankun Liao, Haiquan Qin, Zheng Wang, Linghou Meng, Wentao Wang, Jungang Liu, Xianwei Mo

**Affiliations:** 1 Department of Gastrointestinal Surgery, Division of Colorectal and Anal, Guangxi Medical University Cancer Hospital, No.71, Hedi Road, Qingxiu District, Nanning, 530021 Guangxi Autonomous Region China; 2 Guangxi Clinical Research Center for Colorectal Cancer, Division of Colorectal and Anal, Guangxi Medical University Cancer Hospital, No.71, Hedi Road, Qingxiu District, Nanning, 530021 Guangxi Autonomous Region The People’s Republic of China; 3 Medical Imaging Center, Guangxi Medical University Cancer Hospital, Nanning, 530021 Guangxi Autonomous Region China

**Keywords:** Ultra-low rectal cancer, Total intersphincteric resection, Pedicled greater omental transplantation, Low anterior resection syndrome, Anorectal function

## Abstract

**Background:**

Total intersphincteric resection (ISR) is the ultimate anus-preserving surgery for patients with ultra-low rectal cancer (ULRC), which can result in various degrees of anorectal dysfunction. Known as low anterior resection syndrome (LARS), it seriously affects the postoperative quality of life of patients. The aim of this study was to discuss the value of mesorectal reconstruction with pedicled greater omental transplantation (PGOT) to relieve LARS following total ISR in patients with ULRC, hoping to provide new ideas and strategies for the prevention and improvement of LARS.

**Methods:**

We retrospectively analyzed hospitalization data and postoperative anorectal function of 26 ULRC patients, who were met inclusion and exclusion criteria in our center from January 2015 to February 2022. And combined with the results of anorectal manometry and rectal magnetic resonance imaging (MRI) defecography of some patients, we assessed comprehensively anorectal physiological and morphological changes of the patients after surgery, and their correlation with LARS.

**Results:**

In this study, 26 patients with ULRC were enrolled and divided into observation group (*n* = 15) and control group (*n* = 11) according to whether PGOT was performed. There were no significant differences in surgical results such as operative time, intraoperative blood loss and postoperative complications between the two groups (*P* > 0.05). Postoperative follow-up showed that patients in both groups showed severe LARS within 3 months after surgery, but from the 3^rd^ month after surgery, LARS in both groups gradually began to decrease, especially in the observation group, which showed faster recovery and better recovery, with statistically significant difference (*P* < 0.001). Through anorectal manometry, the mean rectal resting pressure in the observation group was significantly lower than that in the control group (*P* = 0.010). In addition, the postoperative thickness of the posterior rectal mesenterium in the observation group was significantly higher than that in the control group (*P* = 0.001), and also higher than the preoperative level (*P* = 0.018). Moreover, rectal MRI defecography showed that the neo-rectum had good compliance under the matting of greater omentum, and its intestinal peristalsis was coordinated.

**Conclusions:**

ULRC patients, with the help of greater omentum, coordinated their neo-rectum peristalsis after total ISR and recovery of LARS was faster and better. PGOT is expected to be an effective strategy for LARS prevention and treatment of ULRC patients after surgery and is worthy of clinical promotion.

## Introduction

Rectal cancer (RC) is one of the common malignant tumors in the world, with high morbidity and mortality [1]. By far, the primary clinical treatment strategy and possible cure for rectal cancer is surgery, which often depends on the location of the rectal tumor [2]. Clinically, rectal cancer is usually divided into high, middle and low rectal cancer, and even ultra-low rectal cancer (ULRC), according to the height of the rectal tumor from the dentate line, of which low and ultra-low rectal cancer account for about 60%-70% [3]. For surgeons, anus-preserving for ULRC has always been difficult and challenging both technically and in terms of anal function preservation. In the past, abdominoperineal resection (APR) was adopted for ULRC patients. As a result, the anus could not be preserved, and a permanent colostomy was needed, resulting in poor postoperative quality of life [4–6].

In recent years, with the increasing recognition of 10 mm or even 5 mm safe distal margin, and 1 mm circumferential margin [7–11], as well as the promotion of neoadjuvant chemoradiotherapy and the improvement of surgical techniques [12, 13], total intersphincteric resection (ISR) has become the ultimate anus-preserving approach and is accepted by most surgeons [14, 15]. However, in the pursuit of extreme anus-preserving, the pelvic floor structure of patients will be damaged inevitably by surgical trauma, resulting in partial decline or even loss of postoperative anorectal function [16, 17]. More than 80% of patients develop anorectal dysfunction of varying degrees within one year after surgery, which is known as “low anterior resection syndrome (LARS)”. It lasts for a long time, and severe cases can be affected for life [18–20].

There is no standard and effective prevention and treatment of LARS after surgery. In our center, the pedicled greater omentum was applied in the reconstruction of the sacral fascia after total mesorectal excision (TME) in patients with middle and low rectal cancer, and it was observed that the anorectal function of these patients recovered faster and better, with satisfactory results [21]. This inspired us to pay attention to the difference between the expansion characteristics of sigmoid colon or descending colon used to construct the neo-rectum and the physiological characteristics of the original rectum, and to the influence of the changes in the volume, compliance, pressure and movement of the neo-rectum on postoperative anorectal function, which may be an important reason for the occurrence of LARS.

Therefore, we retrospectively analyzed the surgical results and postoperative follow-up results of anorectal function of ULRC patients who underwent total ISR from January 2015 to February 2022 in our center, and combined with the results of anorectal manometry and rectal magnetic resonance imaging (MRI) defecography in some patients to comprehensively evaluate postoperative anorectal changes. The objective of this study was to verify the value of mesorectal reconstruction with pedicled greater omental transplantation (PGOT) to relieve LARS following total ISR in patients with ULRC and provide new ideas and strategies for LARS prevention and treatment after anus-preserving in ULRC patients.

## Methods

### General information

This is a single center retrospective study. The hospitalization data of 26 ULRC patients who met the inclusion and exclusion criteria in the Division of Colorectal and Anal of Guangxi Medical University Cancer Hospital from January 2015 to February 2022 and the postoperative anorectal function results were retrospectively analyzed.

### Inclusion criteria

(1) 18 ≤ Age ≤ 70 years old; (2) Preoperative electronic colonoscopy and rectal MRI were performed to determine the location of the tumor and its distance from the dentate line. The lowest margin of tumors was below peritoneal reflection and within 5 cm above the dentate line, which was diagnosed ULRC; (3) Rectal adenocarcinoma was diagnosed by histopathology before surgery, with or without preoperative neoadjuvant therapy; (4) Distant organ metastasis was excluded by preoperative CT examination, and no intraperitoneal metastasis was found by intraoperative laparoscopic exploration. The clinical stage was I-III (UICC/AJCC, 8th edition [22]); (5) Total mesorectal excision (TME) + total ISR were performed; (6) Sphincter function was normal before surgery, and there was no history of frequent fecal incontinence with fluid or solid stool; (7) The patients were able to complete postoperative anorectal function follow-up.

### Exclusion criteria

(1) Patients with digestive tract reconstruction using staplers; (2) History of other tumors; (3) History of severe cardio-cerebral and vascular diseases; (4) Recurrent rectal cancer; (5) A history of previous abdominal, pelvic, or anal surgery; (6) Patients who received radiotherapy within 1 year after surgery; (7) If a temporary ileostomy was performed after surgery, patients whose stoma was not closed after surgery were excluded from the study.

### Preoperative management

Preoperative doctor-patient communication was carried out, and consent was obtained from all patients and their family members, and surgical consent was signed.

### Total mesorectal excision plus total intersphincteric resection

The steps are as follows: In the first stage, the posterior peritoneum was dissected at the level of the sacral promontory, and then separated along the root of the sigmoid mesangium from bottom to top to complete the dissociation of the sigmoid colon and regional lymph node dissection. Then turn to the anal side to free the rectum and its mesangium, and as far as possible to the anal side to the upper end of the internal rectal sphincter. The next stage was the combined trans-anal operation, from the intersphincter sulcus into the internal and external sphincter space, along the sphincter space to the ventral dissociation, until the two sides of the connected, dragged out of the rectum through the anal. During the operation, the distal and circumferential margins of the tumor were examined for rapid frozen pathological examination to ensure that the external sphincter sparing surgery was performed on the basis of negative surgical margins. After trans-anal removal of the specimen, the digestive tract was reconstructed by manual colon-anal anastomosis. Temporary ileostomy was performed in some patients after surgery, while anal tubes were placed outside the anus to drain in the patients without temporary ileostomy.

### Pedicled greater omental transplantation

**Fig. 1:**
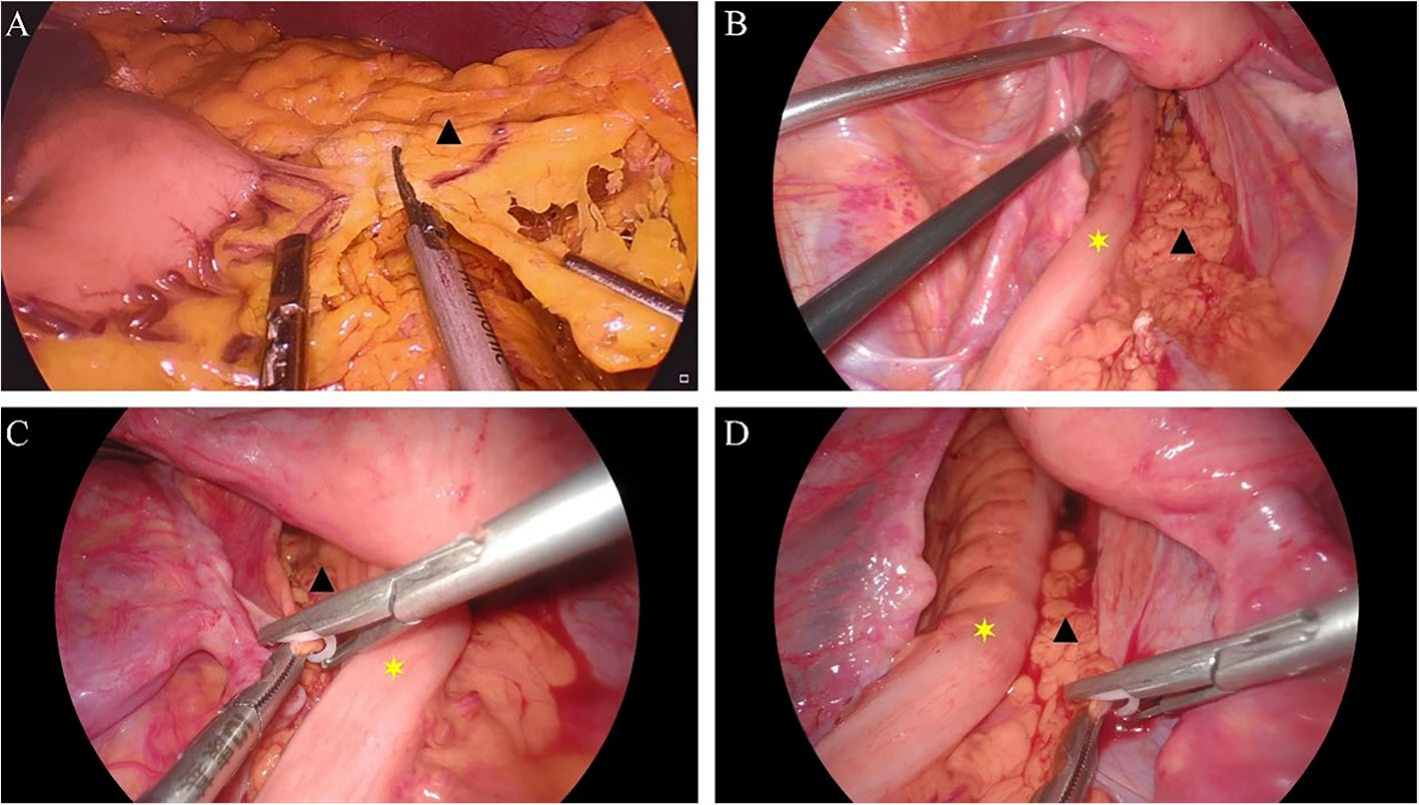
Technical essentials and process of pedicled greater omental transplantation. (**A**) showed clipping of the greater omentum; (**B**) demonstrated the removal of the pedicled omentum into the pelvic cavity, which is fully extended and padded behind and lateral to the neo-rectum; (**C**, **D**) The fixation of the omentum with the bilateral pelvic peritoneum with the Hem-o-lok (yellow asterisk indicated the reconstructed neo-rectum, and black triangle indicated the omentum) was demonstrated

The vessels at the left edge of the omentum were isolated and severed under laparoscopy, and from the left side of the omentum, the omentum was separated to the right along the gastroepiploic vascular arch. During the process of dissociation, attention should be paid to protecting the vessels at the right edge of the omentum. Then, with the right marginal vessels of the omentum as the pedicled axis, the omentum was transferred downward into the pelvic cavity, filled into the posterior rectal space (presacral space), fully extended and padded behind and lateral to the rectum, and the omentum was properly fixed with Hem-O-Lok clips to the pelvic peritoneum on both sides (shown in Fig. 1). During the operation, attention should be paid to avoid distortion of the vascular pedicle to prevent ischemia and necrosis of the omentum.

### Postoperative management

The perianal nursing and rehabilitation guidance of the patients should be done well after surgery. At the same time, the patients were advised to avoid defection control, anal contraction and anal levitation within 2 weeks after surgery, to prevent the separation and retraction of the neo-rectum and the transposition of the transplanted omentum.

### Data collection and analysis

All patients were divided into an observation group and a control group according to whether PGOT was performed or not. The observation group received TME + total ISR + PGOT, while the control group received TME + total ISR. The operation time (min), intraoperative blood loss (ml), postoperative drainage tube removal time (days), postoperative feeding time (days), postoperative length of hospital stays (days), postoperative complications, and temporary ileostomy rate were collected and analyzed. Patients who did not receive temporary ileostomy were followed up every month (1^st^, 2^nd^, 3^rd^, 4^th^, 5^th^, 6^th^ month after operation) and every 3 months after half a year. LARS score [23] was used to evaluate the postoperative anorectal function of the two groups. Patients who received temporary ileostomy were followed up at the above time points starting after the closure of the ileostomy. Thickness of posterior rectal mesenterium (sacral 4 plane in transverse view) was measured by CT or MRI before and after surgery. In addition, the combined anorectal manometry and rectal magnetic resonance imaging (MRI) defecography [24] were used to evaluate the anorectal pressure and morphological structure of some patients, and to comprehensively evaluate the physiological function and morphological characteristics of anorectal after surgery.

### Statistical analysis

Statistical analysis in this study was completed in statistical software SPSS 25.0 (IBM Corp, Armonk, NY, USA). Measurement data were expressed as mean ± standard deviation (x̄ ± s) or median and range. The t test was used for measurement data conforming to normal distribution. If the distribution was skewed, the nonparametric test was used as the statistical method for comparison between groups. The enumeration data were expressed as percentage (%), and the chi-square test (Fisher’s exact test) was used as the statistical method for comparison between groups. The Mann–Whitney rank sum test was used for rank data. Repeated measures ANOVA was used as the statistical method for the comparison of LARS scores between groups. A p < 0.05 was considered statistically significant.

## Results

A total of 32 patients with ULRC were included, after excluding 1 with malignant melanoma, 4 without closure of ileostomy, and 1 with postoperative radiotherapy, 26 patients were finally included in this study. According to whether PGOT was performed or not, 15 patients were divided into the observation group, including 4 males and 11 females, with a median age of 59 (38–75) years old; while there were 11 patients in the control group, including 5 males and 6 females, with a median age of 57 (32–73) years old.

There were no significant differences in age, gender, BMI, underlying diseases, distance between tumor and dentate line, preoperative tumor stage, degree of tumor differentiation, preoperative neoadjuvant therapy, and surgical anesthesia grade between the two groups (*P* > 0.05) (Table 1).

**Table 1:** Baseline characteristics of the two groups

	Observation group (*n* = 15)	Control group (*n* = 11)	*P*
Age (years)	59 (38–75)	57 (32–73)	0.776
Gender			0.419
Male	4	5	
Female	11	6	
BMI (kg/m^2^)	21.23 (17.48–30.76)	24.02 (17.63–32.44)	0.087
Underlying disease			0.689
Yes	5	5	
No	10	6	
Distance between tumor and dentate line (cm)	2.5 (0.3–4.0)	1.9 (0.3–4.8)	0.099
Preoperative tumor stage			0.937
I	2	2	
II	9	6	
III	4	3	
IV	0	0	
Degree of differentiation			0.405
Well differentiated	2	1	
Moderately differentiated	11	10	
Poorly differentiated	2	0	
Preoperative neoadjuvant therapy			0.701
Yes	7	4	
No	8	7	
ASA grade			0.660
I	6	4	
II	8	5	
III	0	0	
IV	1	2	
V	0	0	

*Kg* kilogram, *m* meter, *cm* centimeter *ASA* American Society of Anesthesiologists

### Surgical outcomes

All patients in the two groups underwent laparoscopic surgery successfully, without conversion to laparotomy. All of them were R0 resection, and the degree of surgical resection and dissection were in line with the standard of radical resection of rectal cancer [25].

There were no significant differences in operation time, intraoperative blood loss, postoperative drainage tube removal time, postoperative feeding time, postoperative length of hospital stays and hospitalization costs between the two groups (*P* > 0.05) (Table 2).

**Table 2:** Clinical results of the two groups

	Observation group (*n* = 15)	Control group (*n* = 11)	*P*
Operation time (min)	265 (181–410)	280 (237–420)	0.254
Intraoperative blood loss (ml)	50 (30–200)	100 (15–200)	0.425
Postoperative drainage tube Removal time (days)	5 (4–15)	5 (3–9)	0.689
Postoperative feeding time (days)	3 (1–5)	2 (2–5)	0.702
Postoperative length of hospital stays (days)	7 (5–18)	8 (6–14)	0.383
Hospitalization costs (yuan)	66593.36 (54073.16–85139.12)	70284.10 (49902.22–86810.01)	0.900
Temporary ileostomy			0.109
Presence	7	9	
Absence	8	2	
Postoperative complications			0.683
Within 30 days	4	4	
Yes	11	7	
No			
Clavien-Dindo classification			0.465
I	1	2	
II	3	2	
III	0	0	
IV	0	0	
V	0	0	
Postoperative death within 30 days	0	0	-

*min* minute, *ml* milliliter

A total of 8 patients occurred complications in the two groups within 30 days after surgery, including 4 in each group (Table 3). According to the Clavien-Dindo classification [26], the surgical complications of the two groups were all grade I and II, and no surgical complications of grade III or above occurred. There was no significant difference in surgical complications between the two groups (*P* = 0.683).

**Table 3:** Postoperative complications within 30 days in the two groups

	Observation group (*n* = 15, %)	Control group (*n* = 11, %)
Anastomotic bleeding	0 (0)	1 (9.09)
Peritoneal sepsis	0 (0)	1 (9.09)
Pulmonary infection	1 (6.67)	1 (9.09)
Incomplete ileus^a^	1 (6.67)	1 (9.09)
Neurogenic urinary retention^a^	0 (0)	1 (9.09)
Anemia	2 (13.33)	0 (0)

^a^indicates that multiple complications may occur in the same patient

### Follow-up outcomes

**Fig. 2:**
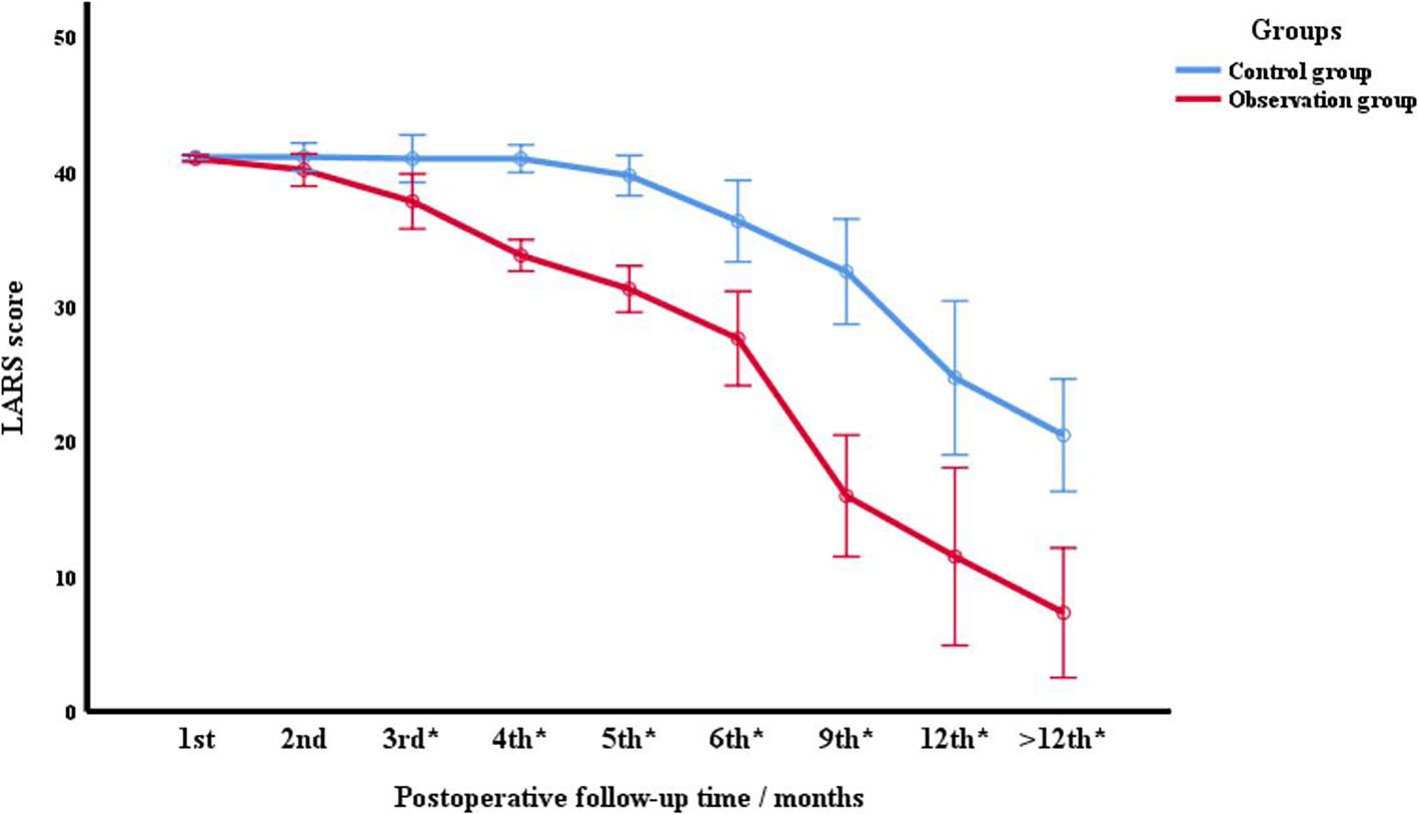
Analysis of the follow-up results of LARS scores in the two groups after surgery. * indicated that the LARS score of the observation group was significantly lower than that of the control group at this time point (*P* < 0.05)

The last follow-up was on June 30, 2022. The LARS scores of the two groups were higher than 30 within 3 months after surgery, and all of them were severe LARS. However, from the 3^rd^ month after surgery, the LARS scores of the two groups gradually decreased, and the anorectal function gradually recovered. Especially 6 months after surgery, LARS value decreased faster and anal function recovered better in both groups. Compared with the control group, the LARS score of the observation group decreased faster with time, and the LARS score of the same time node was relatively lower (*F* = 36.369, *P* < 0.001). There was no significant difference in LARS scores between the two groups at the 1^st^ (*F* = 0.735, *P* = 0.408) and 2^nd^ (*F* = 1.741, *P* = 0.212) months after surgery. At the beginning of 3^rd^ month after surgery [i.e., 3^rd^ (*F* = 6.566, *P* = 0.025), 4^th^ (*F* = 101.431, *P* < 0.001), 5^th^ (*F* = 65.009, *P* < 0.001), 6^th^ (*F* = 16.846, *P* = 0.001), 9^th^ (*F* = 36.935, *P* < 0.001), 12^th^ (*F* = 10.961, *P* = 0.006), and > 12^th^ (*F* = 20.301, *P* = 0.001) month], the mean LARS score of the observation group was significantly lower than that of the control group, and the difference was statistically significant (*P* < 0.05). The LARS score results of the two groups are shown in Fig. 2.

### Anorectal manometry outcomes

A total of 19 patients received anorectal pressure detection after surgery, including 9 patients in the observation group and 10 patients in the control group. The mean rectal resting pressure (RRP), anal resting pressure (ARP), and anal squeeze pressure (ASP) of the two groups were significantly decreased after surgery, and there was no significant difference in other parameters between the two groups (*P* > 0.05) except that the mean RRP of the observation group was significantly lower than that of the control group (*P* = 0.010). The results of anorectal manometry of two groups were shown in Table 4.

**Table 4:** The results of anorectal manometry of two groups

Parameters	Observation group (*n* = 9)	Control group (*n* = 10)	*P*
RRP (mmHg, Mean ± SD)	1.79 ± 0.89	3.50 ± 1.81	0.010
ARP (mmHg, Mean ± SD)	13.34 ± 4.26	10.76 ± 4.10	0.201
ASP (mmHg, Mean ± SD)	14.20 ± 7.74	19.42 ± 13.17	0.332
RAIR (%)			1.000
Presence	7 (77.78)	8 (80)	
Absence	2 (22.22)	2 (20)	
Rectal sensation (ml, Mean ± SD)			
The first sensation volume	25.00 ± 11.95	31.36 ± 7.78	0.071
The discomfort volume	128.75 ± 43.24	132.73 ± 37.71	1.000

*mmHg* millimeter (s) of mercury, *SD* standard deviation, *ml* milliliter, *RRP* rectal resting pressure, *ARP* anal resting pressure, *ASP* anal squeeze pressure, *RAIR* rectoanal inhibitory reflex

### Imaging outcomes

**Fig. 3:**
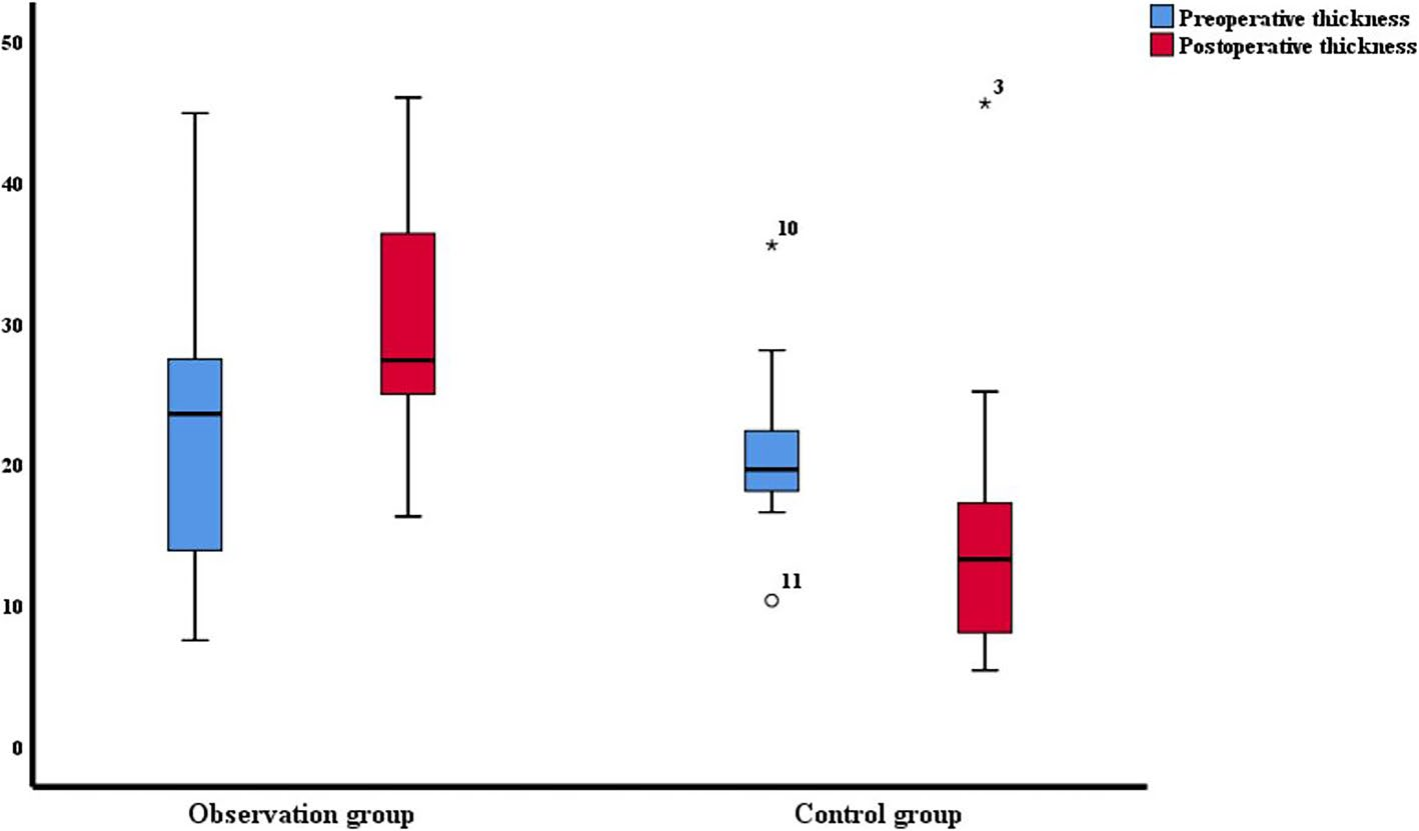
The thickness of the posterior rectal mesenterium before and after surgery in both groups

In terms of thickness of posterior rectal mesenterium (shown in Fig. 3), there was no significant difference between the two groups before surgery (*P* = 0.586). The postoperative thickness of posterior rectal mesenterium of the observation group was significantly higher than that of the control group, and the difference was statistically significant (*P* = 0.001). In the observation group, the thickness of posterior rectal mesenterium of the postoperative subgroup was significantly higher than that of the preoperative subgroup (*P* = 0.018). On the contrary, in the control group, the thickness of posterior rectal mesenterium of the postoperative subgroup was significantly lower than that of the preoperative subgroup (*P* = 0.039).

**Fig. 4:**
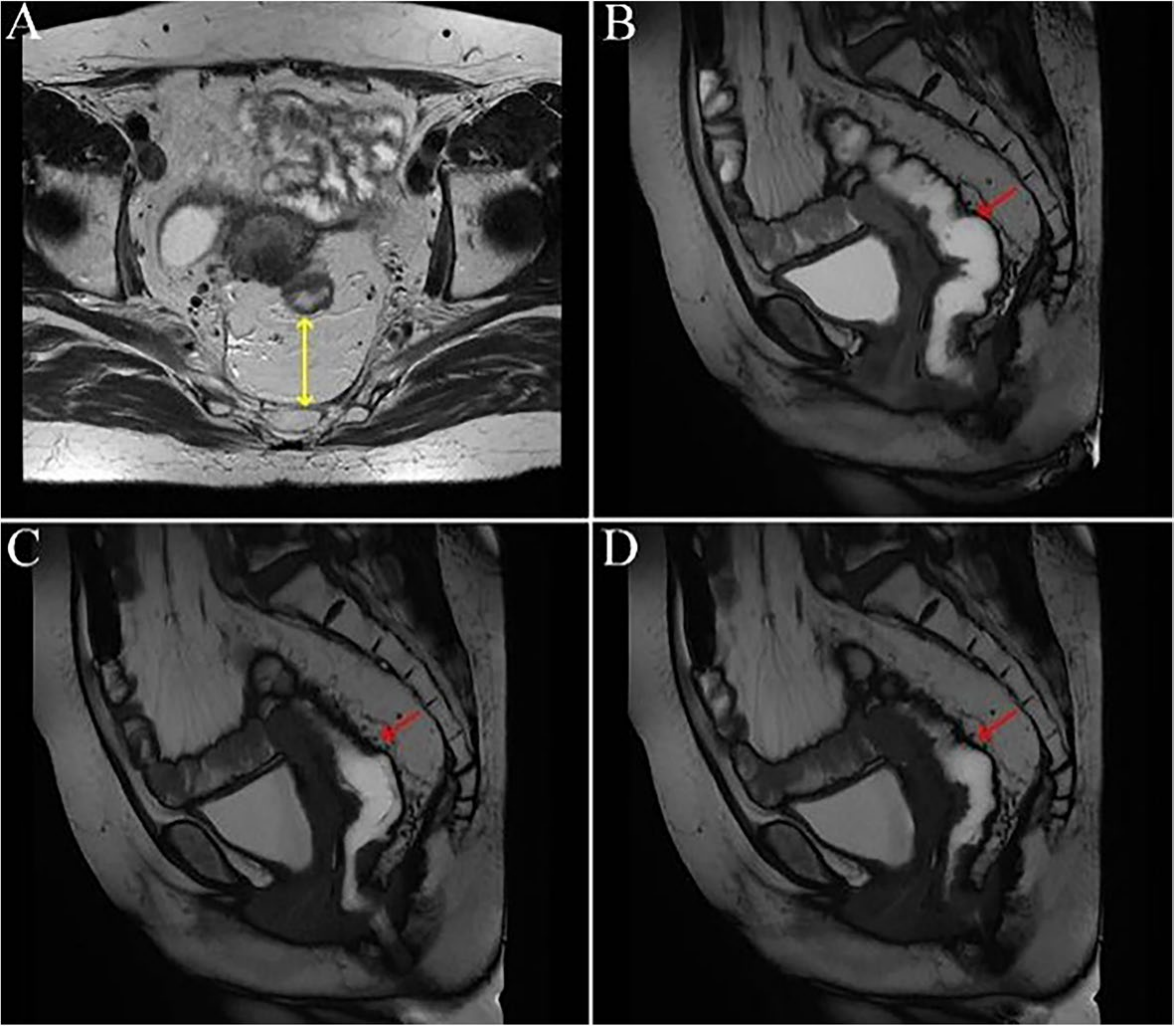
MRI defecography of the rectum in the observation group. **A** showed the cross section of rectal MRI at the sacral 4 level of patients in the observation group (yellow arrow showed the posterior rectal mesangium). **B** → **C** → **D** showed the coordination of neo-rectal peristalsis and the good retraction of the corresponding posterior greater omentum during the force phase of rectal MRI defecography in the observation group (the red arrow showed the posterior rectal mesangium at the sacral 4 level)

**Fig. 5:**
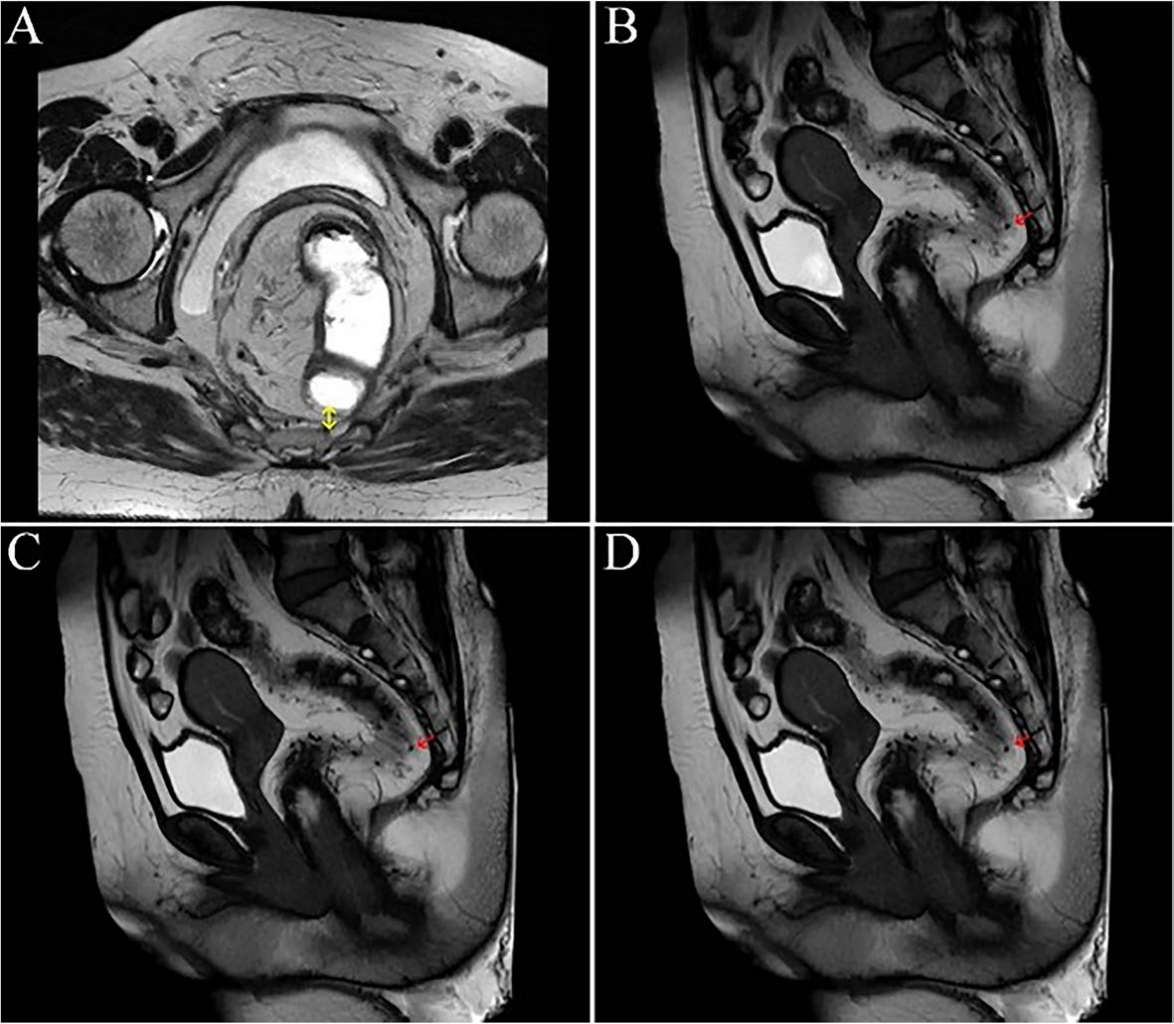
MRI defecography of the rectum in the control group. **A** showed the cross section of rectal MRI at the sacral 4 level of patients in the control group (yellow arrow showed the posterior rectal mesangium). **B** → **C** → **D** showed that the coordination of neo-rectal peristalsis was poor in the control group during force arrangement in rectal MRI defecography (the red arrow showed the posterior rectal mesangium at the sacral 4 level)

Moreover, it could be observed from rectal MRI defecography that the neo-rectum of patients in the observation group showed good compliance and peristalsis coordination with the assistance of the pedicled greater omentum behind the rectum (i.e., anterior sacral) (shown in Fig. 4), but that of the control groups was poor (shown in Fig. 5).

## Discussion

With the progress of anus-preserving technology and the recognition of the optimal and safe distal margin and acceptable tumor results, total ISR has become the ultimate anus-preserving approach [27]. Moreover, long-term studies have shown that complete ISR does not increase the local recurrence rate of patients with ultra-low rectal cancer after surgery compared with APR, and its safety has been recognized by the majority of surgeons [14, 28]. Colon-anal anastomosis was performed manually in both groups. And there were no significant differences in operation time, intraoperative blood loss, postoperative drainage tube removal time, postoperative feeding time, postoperative length of hospital stays and hospitalization costs between the two groups (*P* > 0.05). Except for the control group, the operating methods of the two groups were similar, so there was no significant difference in the surgical results. The surgical approaches were similar in both groups, except that the observation group received an additional PGOT. However, there was no significant difference in operation time between the two groups, which was due to the fact that the patients in the control group were enrolled earlier and the maturity of early total ISR surgery skill was not high, which also prolonged the median operation time of the patients in the control group. In addition, the procedure of PGOT is simple and time-consuming. As a result, there was no significant difference in the median operation time between the two groups.

All patients in the two groups underwent laparoscopic surgery successfully, without conversion to laparotomy. The incidence of postoperative complications was not high, which was similar to that reported by other centers [29, 30]. Moreover, through data review, the postoperative complications of the patients in this study mainly occurred in the early stage of initiating total ISR, and the incidence of postoperative complications in the later stage was relatively low, which may be caused by the improvement of surgical proficiency and surgical skills of the surgeons. In addition, no complications such as omental infection or necrosis were observed in the observation group.

With the application of total ISR, ULRC patients are able to avoid APR and permanent colostomy, but severe anorectal dysfunction will occur after surgery, which also brings low or very low quality of life to the patients. Multiple studies have shown that up to 50%-80% of patients will suffer from anorectal dysfunction of varying degrees after surgery [31–33], which has an impact on their quality of life and may even offset the benefits brought by preserving intestinal continuity [34].

In order to improve defecation function after total ISR, many scholars have done sufficient research and exploration on the physiological function of original rectum, the pathogenesis of LARS, and the reconstruction method of digestive tract [35–38]. However, there is no existing data providing us with direct evidence to improve LARS [39, 40]. In other words, in clinical practice, the choice of digestive tract reconstruction method may be influenced by anatomical and technical possibilities as well as the willingness of the surgeons [39], which also indicates that surgeons need to improve the digestive tract reconstruction method after total ISR.

In the past, our center tried to transplant pedicled greater omentum to the rear of the neo-rectum after TME in patients with middle and low rectal cancer, and the postoperative follow-up found that the defecation function of these patients was better, which provided us with inspiration for improving postoperative LARS [21]. This suggests that we should pay attention to the physiological differences between the dilatation characteristics of sigmoid colon or descending colon used to construct the neo-rectum and the original rectum, and pay attention to the influence of changes in volume, pressure [41], compliance [42, 43] and movement of the neo-rectum on postoperative anorectal function.

On the one hand, postoperative poor defecation function is thought to be associated with the combined effect of smaller neo-rectal volume and higher neo-rectal pressure when anal sphincter function is weakened [44]. The patients in this study had a small volume of the neo-rectum in the early postoperative period, which was confirmed by the decrease in the first sensation volume and the discomfort volume in the anorectal manometry. Due to the small volume of the neo-rectum, the pressure is prone to rise, leading to the loss of the role of the rectal reservoir, and the patient will have frequent defecation even in the presence of a small amount of stool.

On the other hand, postoperative poor defecation function is also believed to be associated with severe pelvic floor tissue adhesion and poor intestinal compliance after digestive tract reconstruction [44, 45]. In order to ensure radical resection, TME is the standard surgical method [46, 47]. However, due to removal of the whole mesorectum, the neo-rectum lacks the matting and buffering of the surrounding mesangium, and the postoperative scar traction caused by pelvic tissue adhesion, the spastic hypermotility of the neo-rectum is easy to lead to increased pressure and decreased compliance. This is also believed to be closely related to the occurrence of LARS [42, 45, 48].

In this study, patients in the observation group received PGOT. Greater omentum is an ideal material for repair. At present, pedicled omental flap, as a source of biomaterials, has been widely used in the prevention or treatment of complications, such as rectovaginal fistula and vesicovaginal fistula, cerebrovascular reconstruction, tissue damage repair, and as a carrier to promote regeneration, etc. [49–52], which has solved many clinical treatment problems. The pedicled omentum was free and transplanted to the pelvic floor to simulate the reconstruction of the mesentery around the neo-rectum and the presacral tissue, which avoided the intestinal stiffness caused by the adhesion between the neo-rectum and the presacral tissue, reduced the pressure of the neo-rectum, improved the compliance of the neo-rectum, and increased the defecation buffer. However, PGOT also has some disadvantages, one of which is the loss of defense, anti-inflammatory, absorption and protection functions of the omentum. Another is whether the pedicle transplantation of the greater omentum will have a certain effect on gastric motility, which needs to be further studied and explored.

Through follow-up of postoperative anorectal function of the two groups of patients, it was found that the intestinal dysfunction of the patients was at the level of severe LARS within 1–3 months after surgery. In addition to the symptoms of postoperative difficulty in defecation (constipation), other patients in both groups had symptoms related to anorectal dysfunction, such as increased frequency of defecation, fecal incontinence, multiple defecation within 1 h, gas-stool confusion, and defecation after eating. This is due to the removal of the rectal ampulla and all the internal anal sphincter during total ISR operation, which destroyed the original physiological structure of the rectum and the anorectal ring [53, 54]. Anal sphincter function, rectal fecal storage capacity and sensory ability of rectal mucosal receptors are important features to maintain fecal abstinence. Anorectal ring also plays an important role in anal self-control function. Therefore, short-term postoperative intestinal dysfunction, defecation and stool control dysfunction are common and serious phenomena after total ISR.

As observed by the LARS curves of the two groups after surgery, although the patients were in a stage of severe LARS within 1–3 months after surgery, the anorectal function of the two groups was slightly improved from the 3^rd^ month after surgery, showing a gradual recovery trend. In addition, the LARS score of the observation group was significantly lower than that of the control group at the time points of 3^rd^, 4^th^, 5^th^, 6^th^, 9^th^, 12^th^ month and > 12^th^ month after surgery, and the difference was statistically significant. It shows that the recovery time and speed of anorectal function in the observation group are better than those in the control group. Furthermore, patients in the observation group showed defecation frequency within 3 months after surgery, and the intestinal function was poor, or some patients showed symptoms of postoperative defecation difficulty (constipation). However, after 3 months of surgery, the patients' defecation function can gradually recover, and their fecal abstinence ability is better. On the 9th month after surgery, most of the patients were in mild LARS, even no LARS in some patients. On the contrary, the recovery of the defecation function in the control group occurred mainly 6 months after surgery, and the overall recovery was still not ideal. Some patients had frequent defecation after 6 months or even 2–3 years after surgery. The difference of postoperative anorectal function recovery between the two groups was earlier and faster in the observation group, which was considered to be related to the low neo-rectal pressure and good improvement of compliance in the observation group under the active assistance of pedicled omentum.

Another important point is that under the matting of pedicled omentum, the thickness of posterior rectal mesangium in the observation group was significantly greater than that in the control group, and also greater than the preoperative level. Accordingly, the mean RRP of patients in the observation group was significantly lower, the neo-rectum of patients in the observation group regained a buffer force, reduced the occurrence of spasmodic movement, significantly reduced RRP, and maintained the pressure gradient formed by ARP and RRP, which played an important role in maintaining the anal self-restraint of patients in the resting state [55]. However, it should not be ignored that some patients show symptoms of constipation and weak defecation in the early postoperative period, which is not only due to the pelvic floor muscle trauma leading to poor active defecation, but also due to low rectal resting pressure, which causes the weakening of intestinal movement. However, about 3 months after surgery, the symptoms of constipation can be improved, which also reflects the recovery of the patient's anorectal function.

In this study, only part of the patients underwent rectal MRI defecography, which resulted in limited reference significance, but it also preliminarily showed the anorectal morphological characteristics of patients after surgery. In the force phase of rectal MRI defecography, the coordination of neo-rectum peristalsis was poor in the control group. In contrast, the greater omentum behind the neo-rectum in the observation group was well scalable, and with its help, the compliance and coordination of the neo-rectum were good.

There were some limitations in this study, which did not fully and comprehensively assess the anorectal function of the patients after surgery. First, the rectal MRI defecography data in the study were usually obtained in the prone position, which may produce poor physiological results compared to the sitting position. Secondly, the sample size of this study was small, which leads to the lack of credibility of the statistical results. Finally, not all of the subjects received anorectal manometry detection and rectal MRI defecography in this study. Although patients' intestinal function was assessed by various methods, the data integrity was insufficient to further analyze postoperative anorectal function and morphological changes.

## Conclusions

Due to the destruction of anorectal physiological structure and surgical trauma, almost all patients with ULRC will have LARS symptoms such as increased defecation times and poor stool control ability after total ISR. Over time, the postoperative anorectal function of patients will recover to a certain extent. Compared with the control group, patients in the observation group were combined with PGOT, and the new rectal compliance of patients in the observation group was better, peristalsis was more coordinated, and the postoperative anorectal function recovery was faster and more ideal.

Therefore, PGOT has a positive effect on LARS after total ISR in ULRC patients and is expected to be an effective strategy for the prevention and improvement of LARS. However, the selection criteria for the implementation of PGOT have not yet been formulated, and the filled omentum has not been quantified and standardized, which needs further study. At the same time, we encourage more clinical centers to join our study.

## Data Availability

The data that support the findings of this study are available from the corresponding author upon reasonable request.
